# Stem carbohydrate dynamics and expression of genes involved in fructan accumulation and remobilization during grain growth in wheat (*Triticum aestivum L*.) genotypes with contrasting tolerance to water stress

**DOI:** 10.1371/journal.pone.0177667

**Published:** 2017-05-26

**Authors:** Alejandra Yáñez, Gerardo Tapia, Fernando Guerra, Alejandro del Pozo

**Affiliations:** 1 Centro de Mejoramiento Genético y Fenómica Vegetal, Facultad de Ciencias Agrarias, PIEI Adaptación de la Agricultura al Cambio Climático (A2C2), Universidad de Talca, Talca, Chile; 2 CRI-Quilamapu, Instituto de Investigaciones Agropecuarias, Chillán, Chile; 3 Instituto de Ciencias Biológicas, Universidad de Talca, Talca, Chile; Institute of Genetics and Developmental Biology Chinese Academy of Sciences, CHINA

## Abstract

The genetic and physiological mechanisms underlying the relationship between water-soluble carbohydrates (WSC) and water stress tolerance are scarcely known. This study aimed to evaluate the main WSC in stems, and the expression of genes involved in fructan metabolism in wheat genotypes growing in a glasshouse with water stress (WS; 50% field capacity from heading) and full irrigation (FI; 100% field capacity). Eight wheat genotypes (five tolerant and three susceptible to water stress) were evaluated initially (experiment 1) and the two most contrasting genotypes in terms of WSC accumulation were evaluated in a subsequent experiment (experiment 2). Maximum accumulation of WSC occurred 10–20 days after anthesis. Under WS, the stress-tolerant genotype exhibited higher concentrations of WSC, glucose, fructose and fructan in the stems, compared to FI. In addition, the stress-tolerant genotype exhibited higher up-regulation of the fructan 1-fructosyltransferase B (*1-FFTB*) and fructan 1-exohydrolase w2 (*1-FEHw2*) genes, whereas the susceptible cultivar presented an up-regulation of the fructan 6-fructosyltransferase (*6-SFT*) and fructan 1-exohydrolase w3 (*1-FEHw3*) genes. Our results indicated clear differences in the pattern of WSC accumulation and the expression of genes regulating fructan metabolism between the tolerant and susceptible genotypes under WS.

## Introduction

Water deficit is an important abiotic stress factor that limits the growth and productivity of major crop species, including wheat [1]. It affects a large number of physiological processes such as leaf gas exchange capacity [2, 3], timing of phenological phases, partitioning and stem reserve utilization, osmotic adjustment, and accumulation of stress-related proteins and antioxidant defense, among others [2, 4]. Also, water deficit influences morphological and agronomic traits such as leaf area, plant height, total biomass, and seed weight [4, 5].

In wheat, water deficit in Mediterranean climates usually occurs from heading and continues during grain formation (i.e. terminal drought), which reduces both the number of kernels per spike, grain weight and yield [6, 7]. Considering this impact on crop productivity, it is essential to identify robust physiological, biochemical and molecular traits that allow selection of water stress-tolerant genotypes for use in breeding programs [1, 8]. Indeed, several studies have reported genotypic variation in the physiological and agronomic traits associated with water stress tolerance in wheat and other cereals [2, 9, 10, 11]. Plants use different mechanisms to tolerate water stress and avoid damage. One of those present in cereals is the accumulation of water-soluble carbohydrates (WSCs) in the stem and leaf sheath up to anthesis, which are then translocated to the spike and grains during grain filling [12, 13]. The accumulation of WSCs begins when the internodes are elongated from the jointing stage to grain filling, however, the total quantities depend on the genotype and environmental conditions [11, 14]. The highest WSC levels are located between the peduncle and penultimate internode [15, 16, 17].

Among the WSCs are glucose (Glu), fructose (Fru), sucrose (Suc), and fructan, the latter being the dominant form in wheat stems [18, 19]. According to Michiels *et al*. [20] wheat genotypes that are able to synthesize and store a higher concentration of WSCs in the stems before anthesis are more likely to exhibit improved grain yield under water stress conditions. This is because the photosynthetic carbon assimilation during post-anthesis is inhibited by water stress conditions, therefore grain growth and filling depend more on stem reserves that are mobilized to the grain [21, 22, 23]. Indeed, translocation of WSCs from the stems could be responsible for 10–20% of the grain yield in irrigated crops [14, 24] and 40–60% under severe water stress conditions during the grain-filling period [21]. Nevertheless, the relationship between stem WSC concentration or content and grain yield (GY) in wheat is not clear, since some studies have found positive relationships [25], but others have reported no significant relationships or even negative relationships [11, 26].

Fructans can account for up to 85% of WSCs in the wheat stem internodes at the stage of maximum accumulation [15, 19, 27], while Suc represents only 10% [28, 29]. Fructans are linear or branched polymers that are synthesized from Suc [30], and they vary in length from trisaccharides (1-ketotriose, 6-ketotriose, and neo-ketotriose) to polysaccharides that have hundreds of Fru units [31]. Fructans, whether linear or branched, are formed by Fru molecules and often by terminal Glu [32]. In wheat, fructans are mixed levans (graminan-type) composed by both (2–1)- and (2–6)-linked β-D-fructosyl units [33]. Genotypic differences in stem WSC concentrations are mainly attributed to fructans [12, 18].

Fructan biosynthesis is mediated by four fructosyltransferase (FT) enzymes [32, 34]: 1-SST (sucrose:sucrose 1-fructosyltransferase); 1-FFT (fructan:fructan 1-fructosyltransferase); 6-SFT (sucrose:fructan 6-fructosyltransferase); and 6G-FFT (fructan:fructan 6G-fructosyltransferase). In wheat, 1-SST produces the trisaccharide, 1-ketotriose (1-K), and Glu from two Suc molecules, 6-SFT uses 1-K as a substrate to produce 1- and 6-ketotriose (1-K and 6-K, a branched tetrasaccharide), and 1-FFT and 6-SFT are involved in chain elongation [12, 35]. The enzyme 6-SFT transfers a Fru unit from a Suc unit to a Fructan by β (2,6) linkages [36]. In the graminae, *Bromus pictus*, the expression of the 6-SFT enzyme is accompanied by accumulation [37], and in *Lactuca sativa*, the increase in plant fructan content is directly related to an increase in the WSC content [38]. In wheat, the expression of the *1-SST* and *6SFT* genes in the stem was positively correlated with stem WSCs and fructan concentrations [12].

The mobilization of stored carbohydrates requires fructan hydrolysis, which is catalyzed by fructan exohydrolase (FEH) enzymes [12]. These include 1-fructan exohydrolase (1-FEH) and 6-fructan exohydrolase (6-FEH) [39, 40, 41] and they catalyze the reaction of fructan, which participates in fructan depolymerization, with β (2,1) and β (2,6) linkages, respectively [42]. The β (2,6) linkages are predominant in wheat stems [35, 43].

As far we are aware, there is little information on how the WSC is accumulated and mobilized from the stem to the grains after anthesis, and how this process is affected by drought stress. Also, the gene expression patterns of stem enzymes responsible for fructan accumulation and remobilization in wheat genotypes growing under water stress and well-irrigated conditions are little understood. A recent study from Zhang *et al*. [26] showed that high expression of the *1-FEH* w3 gene contributed to the high levels of fructan and stem WSC remobilization to the grains in bread wheat under drought conditions. Another study by Cimini *et al*. [34] indicated that the accumulation of fructans in grains of durum wheat growing in field conditions was closely associated with the gene expression and activity of fructan biosynthetic enzymes (mainly 1-SST and 1-FFT).

This study aimed to evaluate the effect of water stress on the concentration of WSCs in stems and the expression of genes involved in fructan metabolism from anthesis to maturity in wheat genotypes with different tolerances to water stress. The genotypes were selected from a large set of 384 cultivars and advanced lines of spring wheat, which were evaluated in the field under water stress and fully irrigated conditions, during two growing seasons [11]. We hypothesize that genotypes with contrasting drought tolerance exhibit different WSC and gene expression dynamics when they are subjected to water deficit.

## Materials and methods

### Plant material and growth conditions

Eight contrasting genotypes were selected according to the yield tolerance index (YTI) from a study conducted under field conditions where water deficit tolerance was evaluated in 384 cultivars and advanced lines of spring wheat [11]. Two experiments were conducted under greenhouse conditions at the Instituto de Investigaciones Agropecuarias (INIA, Institute of Agricultural Research) Quilamapu, Chillán (36°31' S; 71°54' W), Chile in the 2013–2014 and 2014–2015 growing seasons. Eight wheat genotypes (five tolerant and three susceptible to water stress) were evaluated for WSC accumulation in the first experiment (experiment 1) and two contrasting genotypes in terms of grain yield under water stress conditions in field experiments [11] and stem carbohydrate accumulation were evaluated in the second experiment (experiment 2) (Table 1). Greenhouse conditions were 12 h light at 22°C and 55% to 60% relative humidity. Seeds were sown in 5 l pots (4 seeds per pot) in a substrate mixture of loam soil, vermiculite and sand, in a ratio of 5.5: 2.0: 2.5 (v/v), respectively. The physical and chemical properties of the selected soil were: 48 ppm N, 19.91 ppm P, 388.3 ppm K, and pH 6.15. Additionally, at sowing the plants were fertilized with Basacote Plus 3M (COMPO, Münster, Germany), which is a controlled-release fertilizer, and it was applied at a rate of 3 g l^-1^ substrate.

**Table 1:** Genotypes evaluated in experiments 1 and 2, and their level of tolerance to water stress under field conditions, according to the yield tolerance index (YTI).

Genotype	Origin	YTI^1^	Tolerance to stress
**Experiment 1**			
FONTAGRO 8	INIA-Chile	0.60	Tolerant
Pantera	INIA-Chile	0.49	Tolerant
Don Alberto	INIA-Uruguay	0.47	Tolerant
LE2384	INIA-Uruguay	0.43	Tolerant
QUP2522	INIA-Chile	0.35	Tolerant
Fontagro 69	INIA-Chile	0.23	Susceptible
CCCI09	INIA-Uruguay	0.20	Susceptible
Fontagro 98	CIMMYT-Mexico	0.18	Susceptible
**Experiment 2**			
LE 2384	INIA-Uruguay	0.43	Tolerant
Fontagro 69	INIA-Chile	0.23	Susceptible

1: According to del Pozo *et al*. [11]; higher values indicate more tolerance to water stress.

In experiment 1, seeds were sown on 31 July 2013. Two irrigation treatments were established from heading (Zadoks stage Z5.5) [44]: 50% (water stress; WS) and 100% (full irrigation; FI) of field capacity. Before heading, all the plants were grown to field capacity. Fifteen pots were established for each genotype and treatment.

In experiment 2, seeds were sown on 16 July 2014 and the same water treatments as in experiment 1 were established. Soil water content was evaluated by 10HS sensors (Decagon Devices, USA) connected to an EM-50 data logger (Decagon Devices, USA). The 10HS sensor determines volumetric water content by measuring the dielectric constant of the soil using frequency domain capacitance technology. Eight humidity sensors were available, two for each genotype and treatment, and data are shown in S1 Fig.

### Analysis of WSCs, Glu, Fru, Suc, and Fruct

Water-soluble carbohydrates were evaluated in experiments 1 and 2 from anthesis (Z6.5) to physiological maturity (Z9.0). Samples were taken from two primary stems, with four replicates, at different developmental stages from anthesis to maturity, at 10-day intervals in experiment 1 and 7-day intervals in experiment 2. The WS treatment started at heading in each genotype and this was approximately ten days before anthesis (S1 Fig). On each sample date, two stems were cut (between the penultimate and ultimate node) and mixed; half of them were dried at 65°C for 48 h and used for measuring WSCs and the other half were stored at -80°C for quantifying FT and FEH gene expression. The anthrone (Merck, Germany) method was used to determine WSC concentration [45]. The Glu, Fru, Suc, and fructan contents were determined in experiment 2, in the two contrasting genotypes, in terms of WSC content. The powdered samples were extracted in buffer that contained 80% ethanol and 10mM HEPES-KOH pH 7.4 and incubated at 70°C for 2 h with shaking. After centrifugation for 30 min at 10000 rpm at room temperature, the supernatant was stored at -20°C. The pellet was used for further extraction by adding 2000 μl of extraction buffer, incubating at 65°C for 24 h with continuous shaking and centrifuging at 10000 rpm for 15 min. The supernatant was collected and added to the previously stored sample. Samples were clarified using Carrez Reagent 1: 85 mM potassium hexacyanoferrate ferrocyanide, and Carrez Reagent 2: 85mM, zinc sulfate until the metabolites were quantified for 100 samples with the Sucrose, D-Glucose, and D-Fructose K-SUFRG enzymatic assay kit (Megazyme, Ireland) according to the manufacturer’s protocol (K-SUFRG 06/14).

For fructan measurement 100 mg of powder samples were extracted using 40 ml of milli-Q water at 80°C for 20 min. Fructan quantification was performed using the Megazyme Fructan assay kit (Megazyme International, Ireland), according to the manufacturer’s description.

### Physiological traits

Chlorophyll content (SPAD index), relative water content (RWC), and stomatal conductance (gs) in the flag leaves were evaluated in experiment 2 from the anthesis stage (Z6.5) to the start of the grain dough stage (Z8) with four replicates. Flag leaf chlorophyll content was measured with a SPAD 52 portable chlorophyll meter (Minolta Spectrum Technologics Inc., Plainfield, IL, USA). To measure RWC, fresh flag leaf samples were weighed, submerged in distilled water for 24 h at 4°C, and finally dried at 65°C for 48 h. Relative water content was calculated as: RWC = [(FW-DW) x 100] / (TW-DW), where FW, DW and TW are the fresh, dry and turgid leaf weights, respectively. Stomatal conductance (gs) was measured in the flag leaves with a leaf porometer (model SC-1, Decagon Devices, Pullman, WA, USA).

### Agronomic traits

The following traits were evaluated at physiological maturity: plant grain yield (GY), number of kernels per spike (NKS), 1000-grain weight (TKW), number of spikelets per spike (NSS), number of kernels per spike (KPS), and plant dry matter (DW). Evaluations were performed in two plants per pot, and in four replicates.

### Gene expression analyses

The expression of the FT and FEH genes was evaluated by quantitative RT-PCR (qRT-PCR). Extraction of RNA was carried out from wheat stems using the SV Total RNA System kit (Promega, Madison, WI, USA). The quality of RNA was evaluated by denaturing gel electrophoresis and quantification was performed by measuring the absorbance at 260 and 280 nm in a spectrophotometer (EPOCH, Biotek, VT, USA). The cDNA was synthesized using the Superscript reverse transcriptase system (Invitrogen, Carsbad, CA, USA) from 500 ng of total RNA. Specific primers were designed for the genes, *1-SST*, *1-FFTA*, *1-FFTB*, *6-SFT*, *1-FEHw1*, *1-FEHw2*, *1-FEHw3*, and *6-FEH* using Primer Quest and the Oligoanalyzer platform (https://www.idtdna.com/calc/analyzer). The sequences of the primers utilized for amplifying the target and reference (α-tubulin) genes are indicated in S1 Table. Primers were evaluated according to Czechowski *et al*. [46]. Real time PCR was performed using the 5x HOT FIREPol, EvaGreen qPCR Mix Plus Kit (Biotium, USA) in an Eco Real-Time PCR thermocycler (Illumina, USA). All qRT-PCRs were normalized with threshold cycle (Ct) values of the reference gene. The expression variation for the selected genes was estimated as described below. Evaluations were performed at d0, d7, d14 and d21. Samples in subsequent stages could not be obtained due to accelerated senescence of sensitive genotypes. The values are the mean of three biological and two technical replicates.

### Statistical analysis

A factorial experimental design was used to analyze carbohydrates. It combined three factors: genotype (*G*: ‘Fontagro 69’ and ‘LE 2384’), water treatment (*E*: WS and WI), and developmental stage (*S*: days after anthesis; daa). Each experimental unit consisted of a pot with four plants. Two of them were sampled. Analyses of variance (ANOVA) were performed to determine the effects of genotype, environment (water regime), and growth stage (days after anthesis) and their interaction, utilizing the GLM procedure in the SAS package 9.0 [47].

Gene expression was analyzed with the 2^–ΔΔCt^ method described by Yuan *et al*. (2006) [48]. For any particular gene and day after anthesis, the ΔCt values were calculated as the difference between the expression of the target and the reference gene (α-tubulin). The ΔΔCt values were estimated as the difference between the ΔCt determined under WS or FI conditions and the ΔCt measured under FI and at d0. Analysis of yield components was performed by ANOVA and subsequently by Duncan’s test. Additionally, an ANOVA by physiological stage and genotype was performed to evaluate the effect of genotype and water stress treatment. A correction for false positives associated with the p-values estimated from gene expression analyses was performed utilizing the False Discover Rate [49], using the package qvalue for R [50]. Pearson correlations were also performed between the different analyzed variables to identify association patterns. In particular, the relative expression estimates were analyzed along with physiological traits, and carbohydrate contents, from 0 to 21 daa.

Finally, a principal component analysis (PCA) was developed to analyze simultaneously the assessed variables. All the statistical analyses were performed with SAS-JMP software [47].

## Results

### Concentration of carbohydrates in the intermediate stem

**Fig 1:**
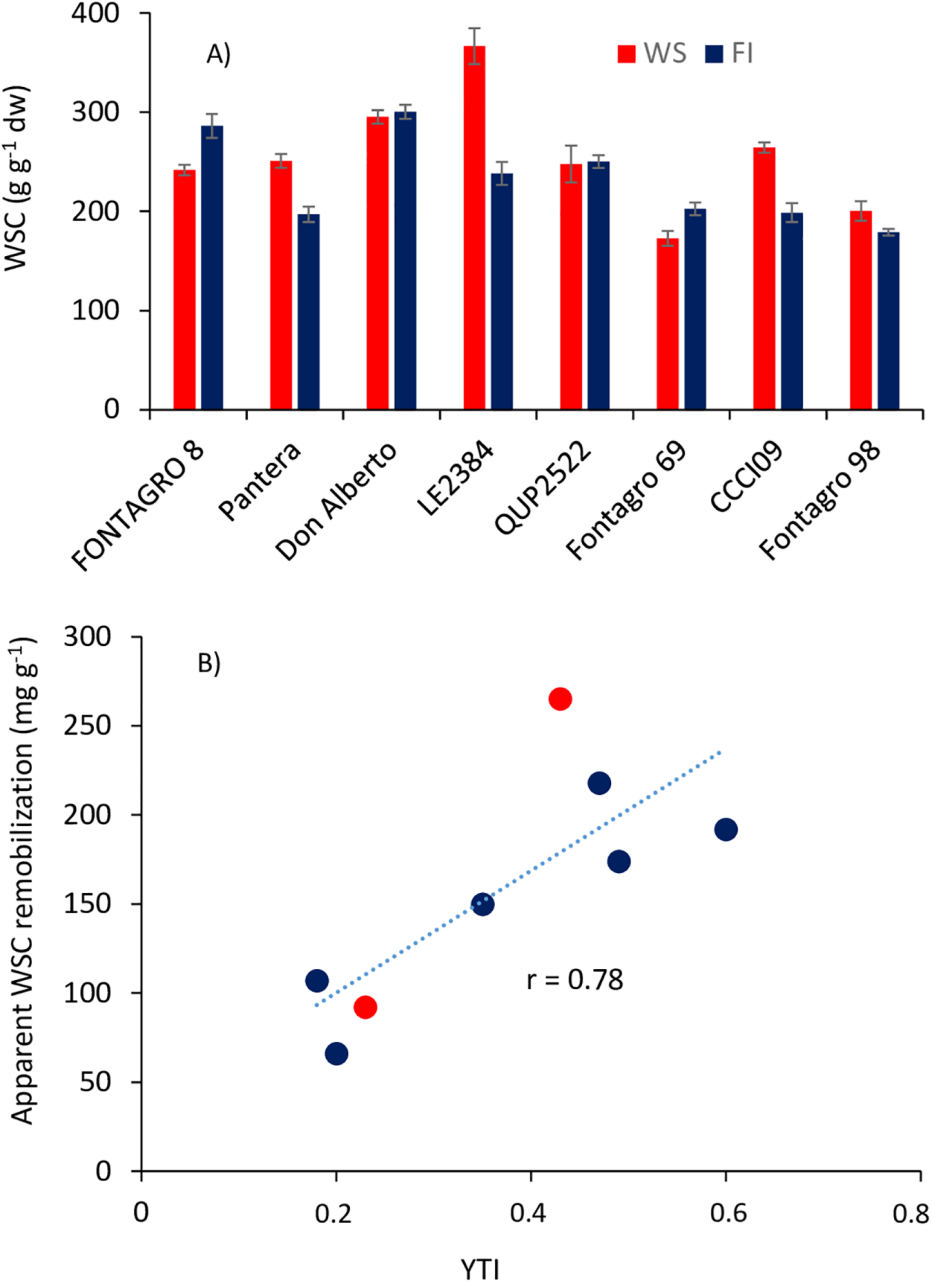
**Maximum water-soluble carbohydrates (WSCs) in the stem (A) and relationship between the apparent WSC remobilization and the yield tolerance index (YTI) determined in field experiments by del Pozo et al. [11] (B)**. WSC was assessed from anthesis to maturity on eight tolerant and susceptible genotypes to water deficit grown under water stress (WS) and full irrigation (FI) conditions (experiment 1). The symbols in red are the two contrasting genotypes used in experiment 2.

A wide variation was observed in experiment 1 for WSCs in the eight genotypes (Fig 1A). The apparent WSC remobilization (DWSC) under WS conditions, estimated as the difference between the maximum and minimum (at physiological maturity, 40 daa), was linearly related to the yield tolerance index, the latter determined in field conditions (Table 1; Fig 1B). Based on this information, the tolerant genotype with maximum WSC concentration (‘LE 2384’) and the susceptible genotype with the lowest WSCs (‘Fontagro 69’) were selected as contrasting varieties (Fig 1B) for studies of carbohydrate composition and gene expression related to fructan synthesis and degradation.

**Fig 2:**
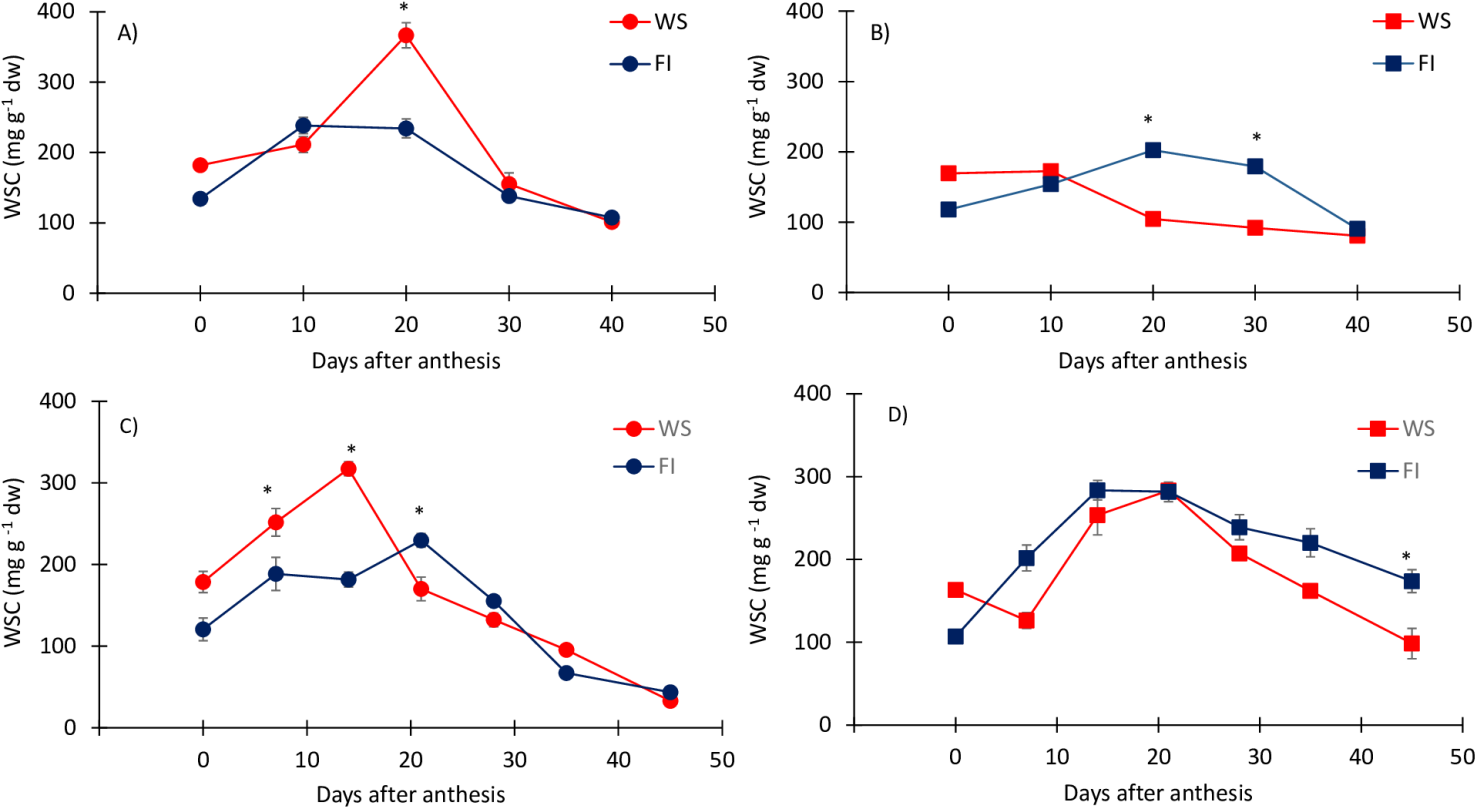
Variation in the concentration of water-soluble carbohydrates (WSCs) in the stem. Measurements were performed from anthesis to maturity on the tolerant (‘LE 2384’; A and C) and susceptible (‘Fontagro 69’; B and D) genotypes, grown under water stress (WS) and full irrigation (FI) conditions, in experiment 1 (A and B) and experiment 2 (C and D). Values are the mean ± SE of four replicates, * P <0.05 (according to Duncan’s test).

In genotype ‘LE 2384’, the stem WSCs under WS conditions were at their maximum at 14–20 daa and significantly higher (P<0.05) than under FI conditions, whereas in ‘Fontagro 69’, the WSCs were significantly higher under FI conditions (Fig 2). Thus, the stem WSCs of both cultivars presented a significant (P<0.001) GxExS interaction (Table 2). In experiment 2, the DWSC under WS conditions was also higher for ‘LE 2384’ (273.76 mg g^-1^; 86.26%) than ‘Fontagro 69’ (188.13 mg g^-1^; 63.25%). This was also true under FI, where the DWSC was 185.92 mg g^-1^ and 109.91 mg g^-1^ for ‘LE 2384’ and ‘Fontagro 69’, respectively.

**Table 2:** Significance levels from ANOVA performed for the glucose, fructose, sucrose, fructan and water-soluble carbohydrate (WSC) concentration in wheat stems measured in experiment 2.

Trait	Genotype (G)	Environment (E)	Stage (S)	GxE	GxS	ExS	GxExS
Glucose	**	***	***	**	***	***	***
Fructose	**	**	***	*	***	*	*
Sucrose	NS	**	***	NS	*	**	NS
Fructan	***	NS	***	***	***	*	**
WSC	***	NS	***	***	***	***	**

* P< 0.05

** P< 0.001

*** P< 0.0001. NS: non-significant.

**Fig 3:**
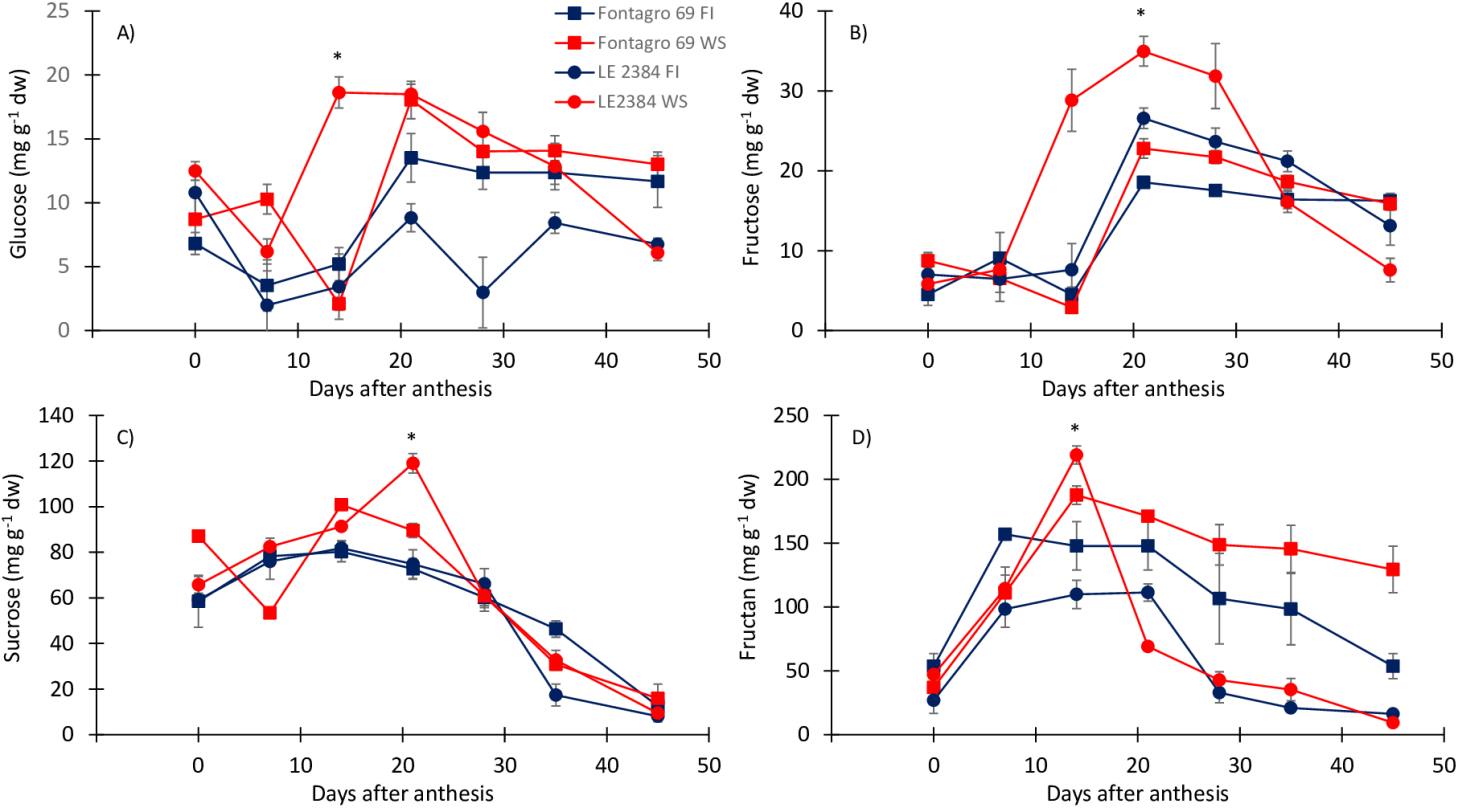
**Changes in concentration of A) glucose, B) fructose, C) sucrose and D) fructans in the stem, from anthesis to maturity.** The tolerant (‘LE 2384’) and susceptible (‘Fontagro 69’) genotypes were grown under water stress (WS) and full irrigation (FI) conditions. Values are the mean ± SE of four replicates, * P <0.05 (according to Duncan’s test).

**Fig 4:**
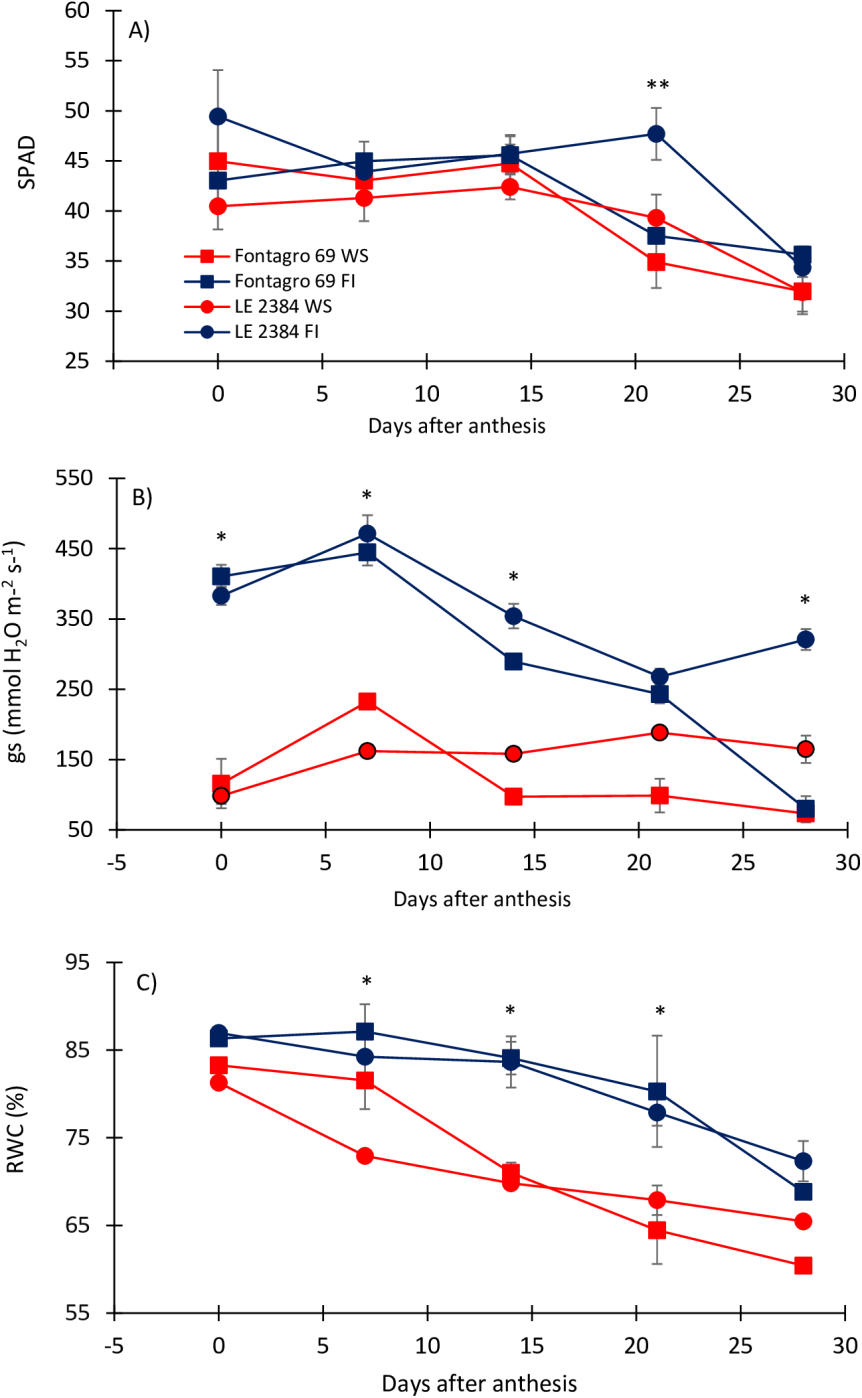
**Changes in A) chlorophyll index (SPAD), B) stomatal conductance (SC), and C) relative water content (RWC) observed from anthesis to maturity**. The tolerant (LE 2384) and susceptible (Fontagro 69) genotypes were grown under water stress (WS) and full irrigation (FI) conditions in 2014 (experiment 2). Vertical lines represent ± SE of the means of the four replicates, *significant differences between treatments, (P<0.05) according to Duncan’s test.

The analysis of Glu, Fru, and fructan concentrations resulted in significant GxExS interactions (P<0.0001, P<0.05, and P<0.01, respectively, Table 2). Water stress increased the concentrations of Glu at 14 daa and Fru between 14 and 28 daa, in genotype ‘LE 2384’ but not in ‘Fontagro 69’ (Fig 3A and 3B). No significant (P>0.05) GxExS interaction was recorded for Suc, however, ‘LE 2384’ had a higher concentration under WS at 21 daa (P<0.05), which explain the significance of the E effect (Fig 3C). Fructans were the predominant WSCs in both cultivars and water conditions, with higher values (P<0.05) at 14 daa under WS for both cultivars, however, they decreased sharply up to 45 daa in ‘LE 2384’ (Fig 4D). As a consequence, ‘LE 2384’ exhibited higher Fructan remobilization under WS conditions (209.65 mg g^-1^) compared with ‘Fontagro 69’ (58.26 mg g^-1^).

### Physiological and agronomical traits

The SPAD index, RWC and gs were evaluated to demonstrate the effect of water deficit in both genotypes. The SPAD index was significantly higher in ‘LE 2384’ under FI at 21 daa (P<0.05) (Fig 4A). The RWC and gs were clearly lower under WS conditions in both genotypes (Fig 4B and 4C). The gs was significantly higher during FI compared with WS treatment. Additionally, gs maintained higher values in ‘LE2384’ under WS (until 28 daa), compared with ‘Fontagro 69’ (Fig 4B).

**Fig 5:**
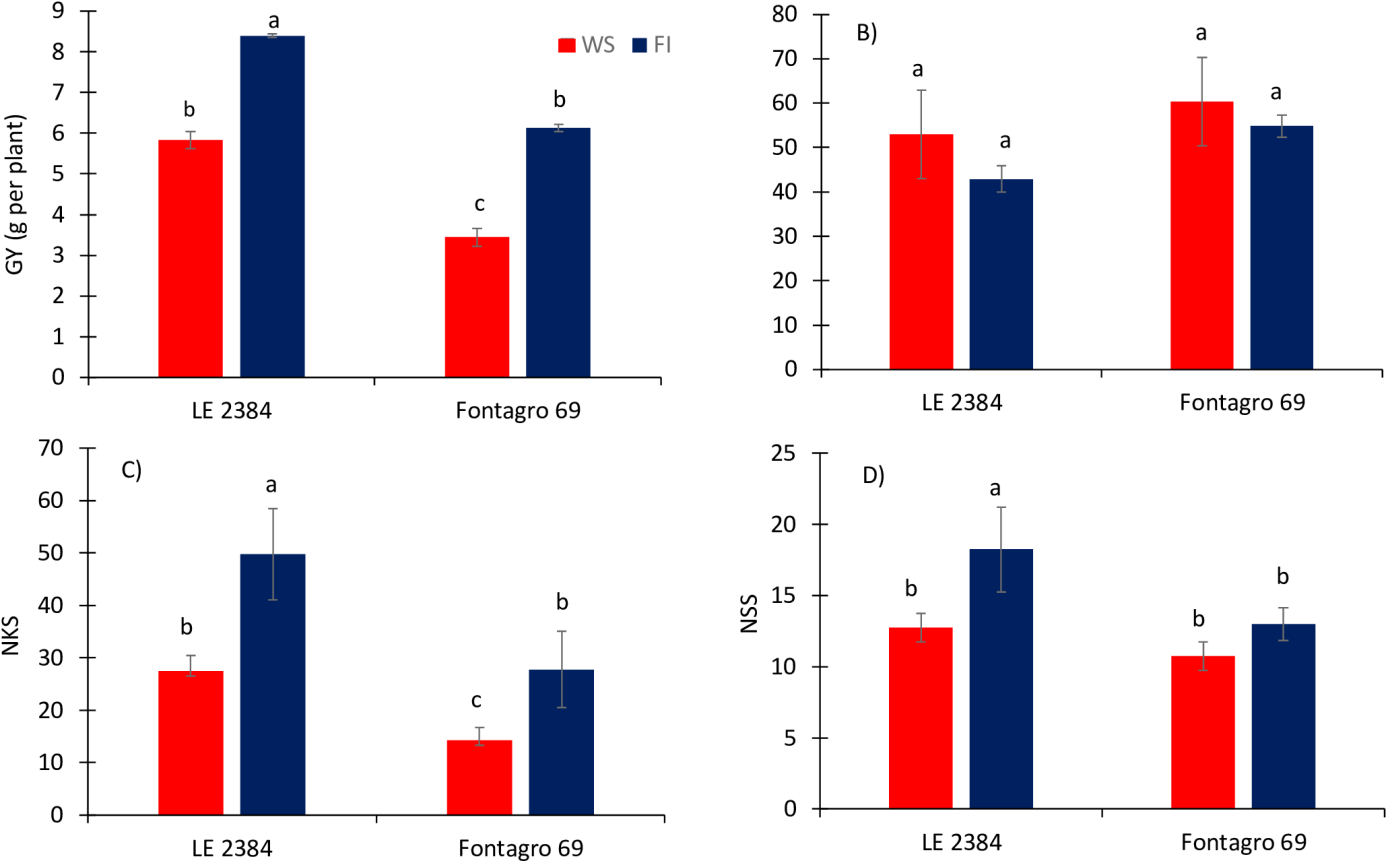
Yield and yield components at physiological maturity for ‘LE 2384’ and ‘Fontagro 69’ genotypes under water stress (WS) and full irrigation conditions (FI). A) grain yield per plant (GY), B) thousand kernel weight (TKW), C) number of kernels per spike (NKS), and D) number of spikelets per spike (NSS). Values are the mean ± SE of four replicates. The lowercase letters above the bars represent significant differences at P<0.05 according to Duncan’s test.

Under WS conditions, GY decreased 30.5% and 43.9% for the genotypes ‘LE 2384’ and ‘Fontagro 69’, respectively (Fig 5A). Water deficit also decreased the number of kernels per spike (NKS) and the number of spikelets per spike (NSS) (Fig 5). Cultivar ‘LE 2384’ had higher GY and NKS under both water regimes (Fig 5A and 5C).

### Analysis of relative gene expression

**Fig 6:**
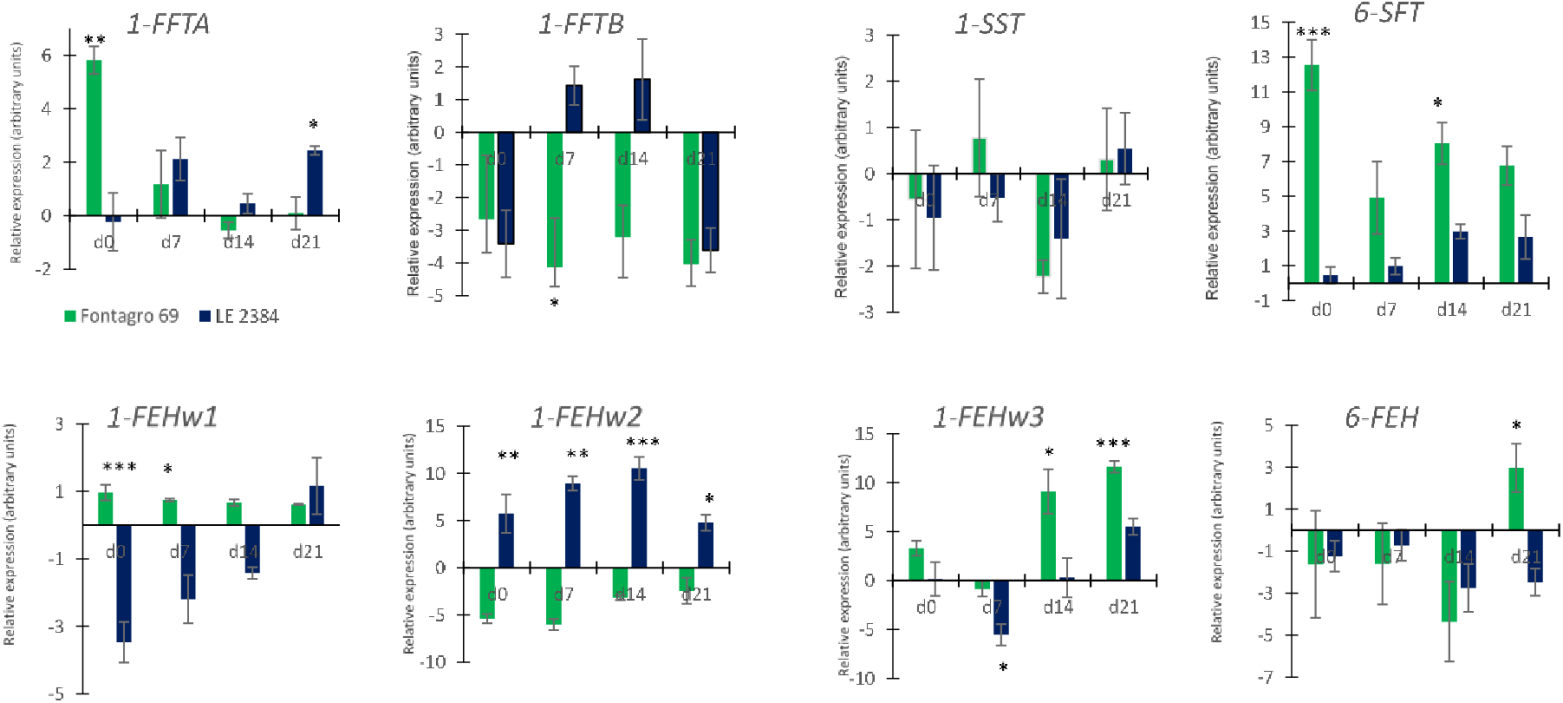
Relative expression of genes involved in fructan metabolism in the stem. The expression of four fructosyltransferase (1-FFTA, 1-FFTB, 1-SST and 6-SFT) and four fructan exohydrolase (1-FEHw1, 1-FEHw2, 1-FEHw3 and 6-FEH) genes was determined in the stems from d0 (anthesis) to 21 days after anthesis (d21), in ‘LE 2384’ and ‘Fontagro 69’ genotypes, grown under water stress (WS) and full irrigation (FI) conditions. Levels of expression were measured by qRT-PCR using primers displayed in Table 2, and the relative expression calculated by 2^–ΔΔCt^ method described in Material and Methods. Vertical lines represent the mean ± SE of three biological replicates, significant differences at P< 0.05*, P<0.001, ** and P<0.0001***.

The determination of relative gene expression was conducted from 0 to 21 days after anthesis. After this period, the plants grown under water stress condition got drier and it was not possible to isolate undegraded RNA. Among the FT genes, *1-FFTA* was significantly up regulated under WS conditions in ‘Fontagro 69’ at d0 and ‘LE 2384’ at 21 daa (P<0.001 and P<0.05, respectively) (Fig 6A). The 1-FFTB gene was down regulated under WS in ´Fontagro 69´ and up regulated in ‘LE 2384’ at 7 and 14 daa (Fig 6B). The expression of the 1-SST gene was similar for both genotypes with small differences between water treatments (Fig 6C). The 6-SFT gene was up regulated under WS in both genotypes, but the level of expression from anthesis to 21 daa was much higher in ‘Fontagro 69’ (Fig 6D).

Three forms of the *1-FEH* gene were evaluated: *1-FEHw1 (1-FEH-6A)*, *1-FEHw2 (1-FEH-6D)*, and *1FEHw3 (1-FEH-6B)*. Results showed significant differences between genotypes in the expression of these genes under WS conditions; in ‘LE 2384’ the *1- FEHw1* gene was down regulated and *1-FEHw2* was up regulated, but the opposite occurred in genotype ‘Fontagro 69’ (Fig 6E and 6F). In addition, the *1-FEHw3* gene was induced in ‘Fontagro 69’ at 14 and 21 daa (Fig 6G). Expression of the *6-FEH* gene was significantly different between genotypes at 21 daa; the susceptible genotype exhibited up regulation while the tolerant genotype exhibited down regulation (Fig 6H). Correction for false positives indicated *q-values* under 0.1 for all the observed significant differences between both genotypes (S2 Table).

Pearson correlations indicated that in genotype ‘LE 2384’, the relative expression of the *1-FFTA* gene was positively correlated with *6-FEH* under WS conditions and with *1-FFTB* under FI (Table 3), whereas in ‘Fontagro 69’, it was positively correlated with *1-FFTB* and *1-SST* under WS, and with *1-FFTB* and *6-SFT* under FI (Table 4). Also, in genotype ‘LE 2384’, the *1-FEHw1* gene was positively correlated with *1-FEHw3* under WS conditions, but negatively with *1-FEHw2* under FI (Table 3). In ‘Fontagro 69’, the *1-FEHw2* was positively correlated with *1-FEHw3* under WS conditions (Table 4). Under WS conditions, fructan was positively correlated with *6-SFT* and *1-FEHw2* genes in genotype ‘LE 2384’, and with *1-FFTA*, *1-FEHw2* and *1-FEHw3* in ‘Fontagro 69’. Under FI conditions, fructan was positively correlated with *1-FEHw1* and *1–FEHw3* in ‘LE 2384’, and with *6-SFT* and *6-FEH* in ‘Fontagro 69’ (Table 4). Fructan presented a high and positive correlation with WSCs in both genotypes and water treatments.

**Table 3:** Pearson correlation matrix between the relative expression of genes encoding fructosyltransferase and exohydrolases enzymes, and water-soluble carbohydrates. Data are from evaluations performed at 0, 7, 14 and 21 days after anthesis, for the genotype “LE 2384” grown under water stress (unshaded matrix) and full irrigation conditions (shaded matrix).

* *	*1-FFTA*	*1-FFTB*	*1-SST*	*6-SFT*	*1-FEHw1*	*1-FEHw2*	*1-FEHw3*	*6-FEH*	*Glu*	*Fru*	*Suc*	*Fruct*	*WSC*
*1-FFTA*		**0,46**	-0,15	-0,17	-0,32	0,37	-0,18	0,35	**0,46**	0,17	-0,06	**-0,53**	-0,36
*1-FFTB*	-0,11		-0,36	-0,37	**0,43**	0,02	0,02	0,25	0,34	0,25	0,19	-0,31	-0,09
*1-SST*	0,25	0,33		**0,45**	0,22	-0,12	**-0,49**	-0,33	**-0,66**	**-0,68**	0,11	-0,05	-0,29
*6-SFT*	-0,31	0,42	0,30		0,37	-0,37	**-0,55**	-0,19	**-0,48**	**-0,78**	0,29	-0,34	**-0,50**
*1-FEHw1*	-0,05	0,11	**-0,50**	0,00		**-0,65**	0,12	0,01	-0,36	0,12	-0,37	**0,87**	**0,62**
*1-FEHw2*	-0,25	0,14	0,11	0,29	-0,14		-0,26	-0,17	0,25	-0,07	0,18	**-0,73**	**-0,55**
*1-FEHw3*	0,25	-0,39	-0,44	-0,23	**0,65**	-0,37		0,20	0,31	**0,66**	-0,39	**0,70**	**0,72**
*6-FEH*	**0,59**	0,00	0,35	-0,30	-0,15	-0,26	0,25		0,43	**0,65**	-0,28	0,39	**0,49**
*Glu*	-0,36	-0,14	0,20	0,10	-0,47	-0,18	-0,24	0,11		**0,79**	-0,11	-0,06	0,28
*Fru*	-0,03	-0,02	0,05	0,25	0,11	-0,39	0,38	0,45	0,29		-0,14	0,36	**0,67**
*Suc*	0,14	0,38	0,35	0,22	-0,34	**0,48**	**-0,59**	-0,07	-0,10	**-0,51**		-0,26	0,02
*Fruct*	-0,34	-0,26	0,26	**0,80**	0,13	**0,58**	-0,33	-0,40	-0,03	0,19	0,10		**0,87**
*WSC*	-0,31	0,32	0,37	**0,85**	-0,01	**0,56**	-0,43	-0,28	0,12	0,25	0,27	**0,95**	

Values in bold face are significant at P<0.05.

*1-FFTA*: fructan 1-fructosyltransferase A, *1-FFTB*: fructan 1-fructosyltransferase B, *1-SST*: sucrose 1-fructosyltransferase

*6-SFT*: sucrose 6-fructosyltransferase, *1-FEHw1*: fructan 1-exohydrolase w1, *1-FEHw2*: fructan 2-exohydrolase w2

*1-FEHw3*: fructan 3-exohydrolase w3, *6-FEH*: fructan 6-exohydrolase, Glu: glucose, Fru: fructose, Suc: sucrose, Fruct: fructan

WSCs: water-soluble carbohydrates.

**Table 4:** Pearson correlation matrix between the relative expression of genes encoding fructosyltransferase and exohydrolases enzymes, and water-soluble carbohydrates. Data are from evaluations performed at 0, 7, 14 and 21 days after anthesis, for the genotype “Fontagro 69” grown under water stress (unshaded matrix) and full irrigation conditions (shaded matrix).

* *	*1-FFTA*	*1-FFTB*	*1-SST*	*6-SFT*	*1-FEHw1*	*1-FEHw2*	*1-FEHw3*	*6-FEH*	*Glu*	*Fru*	*Suc*	*Fruct*	*WSC*
*1-FFTA*		**0,48**	0,35	**0,74**	-0,09	-0,13	-0,33	0,32	0,32	0,38	0,36	**0,89**	**0,86**
*1-FFTB*	**0,63**		**0,47**	0,03	0,37	0,18	-0,17	0,02	0,30	0,29	0,15	0,26	0,29
*1-SST*	**0,52**	**0,55**		0,09	0,04	0,13	-0,38	-0,27	-0,11	-0,44	0,11	0,07	0,02
*6-SFT*	0,37	**0,48**	0,11		-0,33	-0,08	0,01	0,26	0,11	0,22	0,42	**0,72**	**0,70**
*1-FEHw1*	-0,03	-0,10	0,13	0,07		0,03	-0,37	-0,06	0,25	0,08	-0,46	-0,46	-0,44
*1-FEHw2*	0,31	0,09	-0,28	0,22	0,15		0,06	0,05	0,03	0,15	-0,33	-0,23	-0,23
*1-FEHw3*	0,23	0,01	-0,06	-0,22	0,14	**0,74**		-0,43	-0,21	-0,12	0,07	-0,35	-0,30
*6-FEH*	0,23	0,23	0,24	-0,22	0,12	0,32	**0,69**		0,42	**0,73**	0,07	**0,62**	**0,63**
*Glu*	-0,37	-0,22	0,12	-0,14	**0,68**	-0,16	0,21	**0,53**		**0,65**	0,02	0,41	**0,48**
*Fru*	0,13	-0,05	0,09	0,00	0,42	0,36	**0,66**	**0,86**	**0,66**		0,28	**0,50**	**0,61**
*Suc*	0,27	0,56	0,06	**0,50**	-0,08	0,39	0,20	0,21	-0,06	0,13		0,41	**0,56**
*Fruct*	**0,62**	0,28	0,16	0,06	-0,12	**0,55**	**0,56**	0,47	-0,28	0,33	0,41		**0,97**
*WSC*	**0,61**	0,42	0,20	0,33	-0,03	0,46	0,35	0,39	-0,21	0,32	**0,65**	**0,89**	

Values in bold face are significant at P<0.05.

*1-FFTA*: fructan 1-fructosyltransferase A, *1-FFTB*: fructan 1-fructosyltransferase B, *1-SST*: sucrose 1-fructosyltransferase

*6-SFT*: sucrose 6-fructosyltransferase, *1-FEHw1*: fructan 1-exohydrolase w1, *1-FEHw2*: fructan 2-exohydrolase w2

*1-FEHw3*: fructan 3-exohydrolase w3, *6-FEH*: fructan 6-exohydrolase, Glu: glucose, Fru: fructose, Suc: sucrose, Fruct: fructan

WSCs: water-soluble carbohydrates.

**Fig 7:**
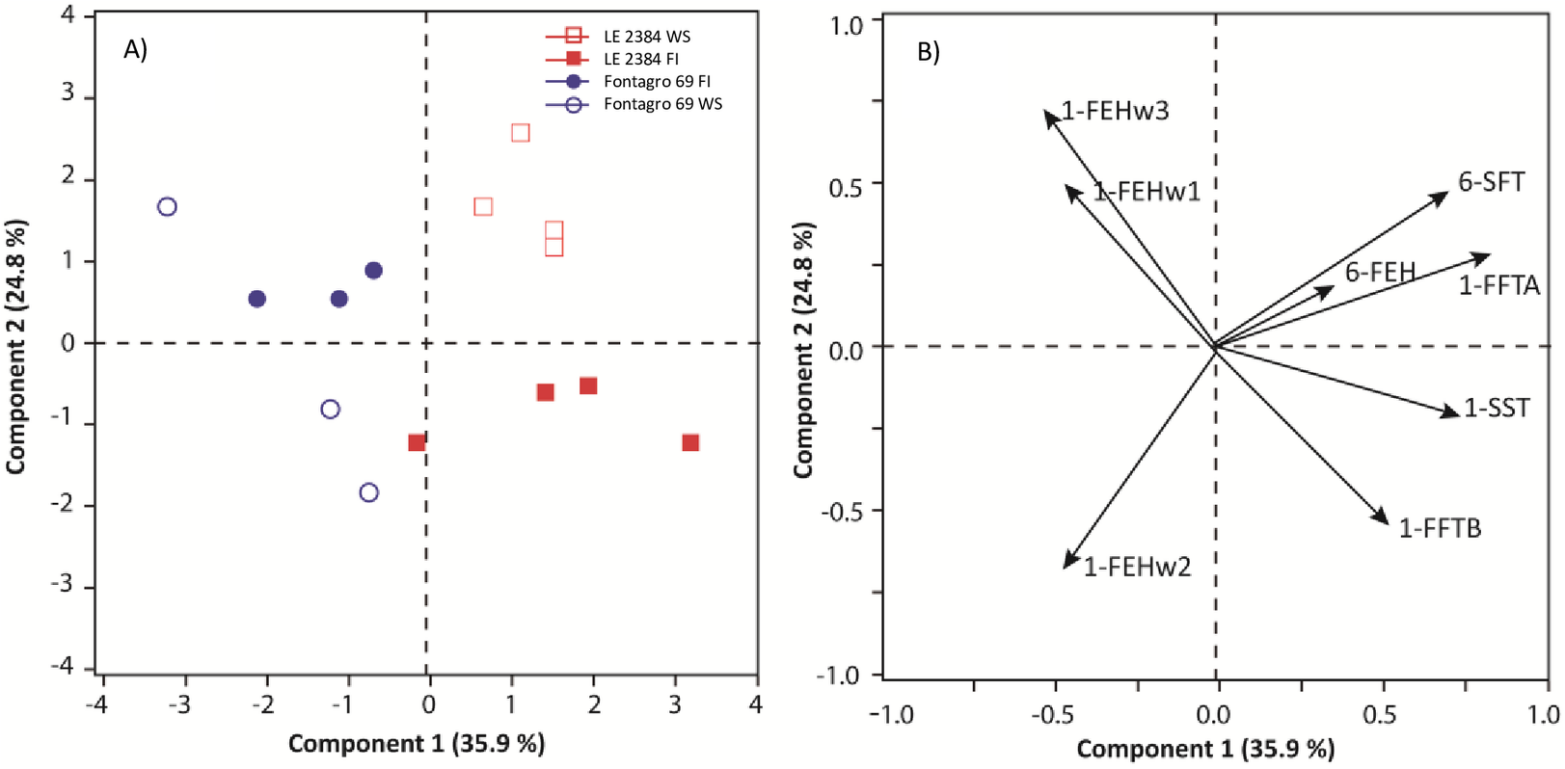
Biplots of the principal component analysis of relative expression of fructosyltransferase and fructan exohydrolase genes in stems. Data are from different days after anthesis (0, 7, 14, and 21), in ‘LE2384’ and ‘Fontagro 69’ genotypes, grown under water stress (WS) and full irrigation (FI) conditions. In A) each point is a sampling date (two sampling dates are superimposed in ´Fontagro 69´). In B) 1-FFTA is fructan 1-fructosyltransferase A; 1-FFTB is fructan 1-fructosyltransferase B; 1-SST is sucrose 1-fructosyltransferase; 6-SFT is sucrose 6-fructosyltransferase; 1-FEHw1 is fructan 1-exohydrolase w1; 1-FEHw2 is fructan 2-exohydrolase w2; 1-FEHw3 is fructan 3-exohydrolase w3; and 6-FEH is fructan 6-exohydrolase.

Principal component analysis revealed a clear differentiation between both genotypes along component 1, and between water regimens along component 2 (Fig 7A). Component 1 explained 35.9% of the variation and was positively and significantly associated with the expression of genes *1-FFTA*, *1-SST* and *6-SFT*. Component 2 explained 24.8% and was associated positively with *1-FEHw3* and negatively with the *1-FEHw2* and *1-FFTB* genes (Fig 7B).

## Discussion

Grain yield and its components were higher in the tolerant genotype (‘LE 2384’) under WS and FI conditions. Furthermore, the reduction in GY caused by water stress was less pronounced in the tolerant genotype (Fig 5). This agrees with what we found under water stress conditions in a Mediterranean environment, where LE 2384 yielded 2.3 and 4.9 t ha^-1^ and ‘Fontagro 69’ yielded 1.0 and 2.5 t ha^-1^, in 2011 and 2012, respectively [11].

Although the two genotypes differed in flowering date the water stress treatment was initiated at the same growing stage (Z5.5), therefore both genotypes were exposed to water stress from heading to maturity and to similar temperature and light conditions in the greenhouse. Indeed, the RWC and gs values, determined in flag leaves during grain growth, clearly indicate that both genotypes were under similar water stress during grain growth (Fig 4). Also, the tolerant genotype had higher gs and RWC values than the susceptible one during the last developmental stages (Fig 4). A number of authors have reported positive relationships between RWC and yield in wheat [51, 52], indicating that RWC can be used as a selection criterion for drought tolerance in cereals [53].

Grain filling is the final growth stage of cereals and is a crucial stage for economic performance. During this time the WSCs stored in the wheat stem are mobilized and converted into grains [14, 18, 29, 54, 55]. Under water stress conditions, mobilization of WSCs and fructan from the stem to the grain is vital for GY because they can compensate for the negative effect of reduced grain production [14, 15, 26, 27]. Our results indicate that under water stress conditions the tolerant genotype was able to accumulate and remobilize more WSCs and Fructans than the susceptible one. The highest mobilization efficiency recorded for the genotypes under WS conditions, especially ‘LE 2384’, could be mainly due to the reduction in high and low molecular weight fructans in the stem [56, 57].

Fructans are the sugars that predominated in the stems during grain growth. Under WS conditions, the highest concentration of fructans was reached at 14 daa (Fig 3). Another study [58] has also reported maximum values between 8–16 daa. Significant differences in Glu and Fru stem concentrations were observed between the two genotypes; under WS conditions the tolerant genotype had higher concentrations, with their maximum occurring at an earlier stage of grain growth, and they had larger differences with respect to FI (Fig 3). The maximum Suc concentration under WS conditions occurred after (tolerant genotype) or simultaneously (susceptible genotype) with the fructan peak (Fig 3). These results suggest that after anthesis fructans can be converted to Suc, which can effectively compensate for the low photosynthetic supply under water stress conditions, and in this way sustain the grain-filling rate [59].

The results of gene expression showed that the tolerant and susceptible genotypes have differential mechanisms to respond to WS (Tables 3 and 4; Fig 6). In the tolerant genotype ‘LE 2384’, the higher up regulation of fructosyltransferase genes (*1-FFTA* and *1-FFTB*) after anthesis under WS seems to be very important. Also, the up regulation of *6-SFT* increased in ‘LE 2384’ as grain filling progressed, and it was positively correlated (r = 0.80) with the fructan concentration in the stem (Table 3; Fig 6). In contrast, ‘Fontagro 69’ exhibited greater levels of up regulation of the *1-FFTA* gene at anthesis (d0) and also a high correlation with fructan under WS conditions (Table 4). The down regulation of *1-FFTB* in the susceptible cultivar could be related to the catalyzation of fructan synthesis in wheat; Li *et al*. [57] had previously found that *1-SST*, *1-FFTA*, and *1-FFTB* could be responsible for this process. This could explain the lower fructan content found in the susceptible cultivar.

The fructan exohydrolase enzymes in wheat are responsible for the degradation of fructan into Fru and Suc when the carbohydrate supply is lower than the demand [23, 60]. High expression levels of the *1-FEH* isoform are associated with graminan degradation, which is necessary to maintain the carbon flow required for grain filling [61], [62]. Under WS conditions, the relative expression of the *1-FEHw2* gene in genotype ‘LE 2384’ was positively correlated with Suc (Table 3), and in ‘Fontagro 69’, the *1-FEHw2* and *6-FEH* genes were positively correlated with Fru (Table 4).

The principal component analysis showed a clear separation between the two cultivars. The tolerant genotype (‘LE 2384’) was associated with the relative expression level of *1-FEHw2*, whereas ‘Fontagro 69’ was associated with the expression of the *1-FEHw1* and *1-FEHw3* genes. Therefore, the high upregulation of *1-FEHw2* in the tolerant genotype could be associated with the carbon flow required at grain filling under WS conditions (Fig 6). Also, higher up regulation of the *1-FEHw3* gene was observed in the susceptible genotype (‘Fontagro 69’), but its expression level in ‘LE 2384’ increased at 21 daa, and probably continued increasing under water stress beyond 21 days after anthesis due to the genotype’s late phenology (Fig 6). A study conducted in two wheat cultivars with high yield and stem WSC content revealed that the cultivar with the greatest expression of the *1-FEHw3* gene presented accelerated remobilization of stem WSCs under water stress conditions [61]. The *6-FEH* gene was down regulated under WS conditions in both genotypes, except in ‘Fontagro 69’at 21 daa. According to Dreccer *et al*. [23] and Chen *et al*. [29], *6-FEH* is not inhibited by Suc and this suggests that it might not be involved in mobilizing the reserves, although earlier studies suggested that the most important enzyme in the stem under WS conditions is *6-FEH*.

In conclusion, the wheat genotypes ‘LE 2384’ and ‘Fontagro 69’, which have contrasting tolerance to water stress, have different physiological and genetic mechanisms to deal with drought conditions. Our results suggest that the selection of genotypes with higher fructan and WSC remobilization efficiencies would lead to cultivars with higher grain yield under WS conditions. The *1-FFTA*, *1-FFTB* and *1-SST* genes influence fructan synthesis, with a differential regulation under water stress. In relation to the *FEH* genes, the expression of the three *1-FEH* members that were assessed seems to depend on the specific mechanisms used by each genotype to deal with water stress. The *1-FEHw2* gene was possibly associated with sugar translocation from the stem to the grains in the tolerant genotype. Further studies, for example wide genome association analyses or the functional evaluation of candidate genes, will complement our current knowledge and support the development of selection tools for improving productivity under the projected climate change scenarios.

## Supporting information

S1 Fig Soil volumetric water content (m^3^ m^-3^) in water stress (WS) and full irrigation (FI) in genotype A) ‘LE 2384’ and B) Fontagro 69. Values are the mean of two sensors (replicates). The arrows indicate the anthesis for each genotype.
(DOCX)

S1 Table Primer sequences used in qRT-PCR for the target and reference genes in experiment 2. Parameter values were determined by the Oligoanalyzer platform (https://www.idtdna.com/calc/analyzer).
(DOCX)

S2 Table False discovery ratio for p-values estimated from gene expression analyses performed to compare contrasting genotypes. (DOCX)

## Abbreviations

1-FEH: 1-fructan exohydrolase
1-FFT: fructan:fructan 1-fructosyltransferase
1-SST: sucrose:sucrose 1-fructosyltransferase
6-FEH: 6-fructan exohydrolase
6G-FFT: fructan:fructan 6G-fructosyltransferase
6-SFT: sucrose:fructan 6-fructosyltransferase
DW: dry weight
FEH: fructan exohydrolase
FI: full irrigation
Fru: fructose
Glu: glucose
gs: stomatal conductance
GY: grain yield
KPS: number of kernels per spike
NKS: number of kernels per spike
NSS: number of spikelets per spike
RWC: relative water content
Suc: sucrose
TKW: 1000-grain weight
WS: water stress
WSC: water-soluble carbohydrates
