# Supplementary material for: Stem carbohydrate dynamics and expression of genes involved in fructan accumulation and remobilization during grain growth in wheat (Triticum aestivum L.) genotypes with contrasting tolerance to water stress
Source: PLoS One. 2017 May 26;12(5):e0177667. doi: 10.1371/journal.pone.0177667 (PMC5446126; doi:10.1371/journal.pone.0177667)
Supplement: S1 Fig — Soil volumetric water content (m3 m-3) in water stress (WS) and full irrigation (FI) in genotype A) ‘LE 2384’ and B) Fontagro 69. Values are the mean of two sensors (replicates). The arrows indicate the anthesis for each genotype. (DOCX) [file pone.0177667.s001.docx]

**S1 Fig.** Soil volumetric water content (m^3^ m^-3^) in water stress (WS) and full irrigation (FI) in genotype A) ‘LE 2384’ and B) Fontagro 69. Values are the mean of two sensors (replicates). The arrows indicate the anthesis for each genotype.
