# Supplementary material for: Stem carbohydrate dynamics and expression of genes involved in fructan accumulation and remobilization during grain growth in wheat (Triticum aestivum L.) genotypes with contrasting tolerance to water stress
Source: PLoS One. 2017 May 26;12(5):e0177667. doi: 10.1371/journal.pone.0177667 (PMC5446126; doi:10.1371/journal.pone.0177667)
Supplement: S1 Table — Parameter values were determined by the Oligoanalyzer platform (https://www.idtdna.com/calc/analyzer). (DOCX) [file pone.0177667.s002.docx]

**S1 Table**. Primer sequences used in qRT-PCR for the target and reference genes in experiment 2. Parameter values were determined by the Oligoanalyzer platform (<https://www.idtdna.com/calc/analyzer>).

| Primers | Sequence (5’-3’) | Primer length  (nucleotides) | Amplicon length (bp) | GC content (%) | Tm  (°C) |
| --- | --- | --- | --- | --- | --- |
| 1-FFTA-F | AGCACCAGCTAGTCTACCTATC | 22 | 108 | 50 | 62 |
| 1-FFTA-R | TCACCCGCAACTGATTTACAA | 21 |  | 42.9 | 63 |
| 1-FFT B-F | CCTTCAGTCGATTCCGAGAAC | 21 | 110 | 52.4 | 62 |
| 1-FFTB-R | TGAGATCGGTGGTGTTGATG | 20 |  | 50 | 62 |
| 6-FEH-F | CAACGTGTACAAGCCGTCATA | 21 | 101 | 47.6 | 62 |
| 6-FEH-R | CCACTACCGAATGGTCAATCAA | 22 |  | 45.5 | 62 |
| 6-SFT-F | CGCTTGCAGAACAGAAACATC | 21 | 108 | 47.6 | 62 |
| 6-SFT-R | CCACCCACCCAAACTATATTGA | 22 |  | 45.5 | 62 |
| 1-SST F | CGACTCTGCCTATCACTTCATT | 22 | 94 | 45.5 | 62 |
| 1-SSTR | TCAACATCATAGCCCTGTCATC | 22 |  | 45.5 | 62 |
| 1-FEHw1-F | GCCAGAGGAAGTGCTAGTATG | 21 | 92 | 52.4 | 62 |
| 1-FEHw1-R | ACAGTGGAGCAAACATGGA | 19 |  | 47.4 | 62 |
| 1-FEHw2-F | GTTGTGTTCCCTGAATATGTCC | 22 | 82 | 45.5 | 61 |
| 1-FEHw2-R | TGCTTTGCCCAACTGAATG | 19 |  | 47.4 | 61 |
| 1-FEHw3-F | TATACTCCCTCCGTCCCAAA | 20 | 102 | 50 | 62 |
| 1-FEHw3-R | TAAATACTCCCTCCGTCCCA | 20 |  | 50 | 62 |
| 1-FEHw2-F | CTTCCATAGTTGTGTTCCCTGA | 22 | 91 | 45.5 | 62 |
| 1-FEHw2-R | ATGCTTTGCCCAACTGAATG | 20 |  | 45 | 62 |
| α-Tub-F | GAGCACACTGATGTCTCTATCC | 22 | 102 | 50 | 62 |
| α-Tub-R | CCTGTTGAGGTTGGTGTATGT | 21 |  | 47.6 | 62 |

GC: Guanine and cytosine, Tm: melting temperature.
