# Supplementary material for: Stem carbohydrate dynamics and expression of genes involved in fructan accumulation and remobilization during grain growth in wheat (Triticum aestivum L.) genotypes with contrasting tolerance to water stress
Source: PLoS One. 2017 May 26;12(5):e0177667. doi: 10.1371/journal.pone.0177667 (PMC5446126; doi:10.1371/journal.pone.0177667)
Supplement: S2 Table — (DOCX) [file pone.0177667.s003.docx]

**S2 Table.** False discovery ratio for p-values estimated from gene expression analyses performed to compare contrasting genotypes.

| **Gene** | **DAS^1^** | **p-value^2^** | **q-value^3^** |
| --- | --- | --- | --- |
| *1-FFTA* | 0 | 0.0025 | 0.0032 |
|  | 7 | 0.4681 | 0.1672 |
|  | 14 | 0.0641 | 0.0407 |
|  | 21 | 0.0328 | 0.0221 |
| *1-FFTB* | 0 | 0.2876 | 0.1370 |
|  | 7 | 0.0153 | 0.0109 |
|  | 14 | 0.0101 | 0.0089 |
|  | 21 | 0.4051 | 0.1672 |
| *1-SST* | 0 | 0.4681 | 0.1672 |
|  | 7 | 0.3445 | 0.1514 |
|  | 14 | 0.3445 | 0.1514 |
|  | 21 | 0.4681 | 0.1672 |
| *6-SFT* | 0 | 0.0025 | 0.0032 |
|  | 7 | 0.1892 | 0.0940 |
|  | 14 | 0.0025 | 0.0032 |
|  | 21 | 0.0153 | 0.0109 |
| *1-FEHw1* | 0 | 0.0025 | 0.0032 |
|  | 7 | 0.0025 | 0.0032 |
|  | 14 | 0.1892 | 0.0940 |
|  | 21 | 0.1892 | 0.0940 |
| *1-FEHw2* | 0 | 0.0025 | 0.0032 |
|  | 7 | 0.0025 | 0.0032 |
|  | 14 | 0.0025 | 0.0032 |
|  | 21 | 0.0065 | 0.0068 |
| *1-FEHw3* | 0 | 0.0867 | 0.0522 |
|  | 7 | 0.0101 | 0.0089 |
|  | 14 | 0.0153 | 0.0109 |
|  | 21 | 0.0025 | 0.0032 |
| *6-FEH* | 0 | 0.4681 | 0.1672 |
|  | 7 | 0.4681 | 0.1672 |
|  | 14 | 0.1892 | 0.0940 |
|  | 21 | 0.0041 | 0.0047 |

^1^) DAS: days after stress

^2^) p-value from Wilcoxon test for two samples (Yuan *et al*., 2006)

^3^) q-value determined by False Discover Rate (Storey and Tibshirani, 2003), using qvalue (Bass et al., 2015)

**References**

Bass A, Dabney A, Robinson D 2015. qvalue: Q-value estimation for false discovery rate control. R package version 2.6.0.

Storey, J., Tibshirani, R. 2003. Statistical significance for genomewide studies. PNAS 100 (16): 9440-9445.

Yuan YS, Reed A, Chen F, Stewart CN (2006) Statistical analysis of real-time PCR data. BMC Bioinformatics 7, 85.
